# Two novel likely pathogenic variants of *HARS2* identified in a Chinese family with sensorineural hearing loss

**DOI:** 10.1186/s41065-020-00157-7

**Published:** 2020-11-24

**Authors:** Jing Yu, Wei Jiang, Li Cao, Xiaoxue Na, Jiyun Yang

**Affiliations:** 1 College of Medical Technology, Chengdu University of Traditional Chinese Medicine, Chengdu, Sichuan 610071 PR China; 2 The Key Laboratory for Human Disease Gene Study of Sichuan Province , Prenatal Diagnosis Center, Sichuan Provincial People’s Hospital, the University of Electronic Science and Technology of China, The First Ring Road West Section 2 #32, Chengdu, Sichuan 610071 PR China; 3 School of Medicine, University of Electronic Science and Technology of China, Chengdu, Sichuan 610071 PR China

**Keywords:** *HARS2*, Perrault syndrome, Next-generation sequencing

## Abstract

Mutations in *HARS2* are one of the genetic causes of Perrault syndrome, characterized by sensorineural hearing loss (SNHL) and ovarian dysfunction. Here, we identified two novel putative pathogenic variants of *HARS2* in a Chinese family with sensorineural hearing loss including two affected male siblings, c.349G > A (p.Asp117Asn) and c.908 T > C (p.Leu303Pro), through targeted next-generation sequencing methods. The two affected siblings (13 and 11 years old) presented with early-onset, rapidly progressive SNHL. The affected siblings did not have any inner ear malformations or delays in gross motor development. Combined with preexisting clinical reports, Perrault syndrome may be latent in some families with non-syndromic deafness associated with *HARS2* mutations. The definitive diagnosis of Perrault syndrome based on clinical features alone is a challenge in sporadic males, and preadolescent females with no signs of POI. Our findings further expanded the existing spectrum of *HARS2* variants and Perrault syndrome phenotypes, which will assist in molecular diagnosis and genetic counselling of patients with *HARS2* mutations.

**Supplementary Information:**

The online version contains supplementary material available at 10.1186/s41065-020-00157-7.

## Introduction

The *HARS2* gene is mapped to chromosome 5q31.3, contains 13 exons and spans approximately 7.9 kb. The *HARS2* gene encodes the highly conserved mitochondrial histidyl tRNA synthetase, which is involved in mitochondrial protein translation [1, 2]. In 2011, Mutations in *HARS2* were first identified as a cause of Perrault syndrome by genome-wide linkage analysis and candidate gene sequencing [1]. Perrault syndrome is an autosomal recessive disorder with main clinical features of bilateral SNHL, a mild to profound degree of hearing loss, and ovarian dysgenesis in females. When the onset of moderate SNHL is in early childhood, it may present as progressive hearing loss. Ovarian dysfunction ranges from primary amenorrhea to primary ovarian insufficiency (POI), which can lead to infertility. Affected males, on the other hand, show normal pubertal development and are typically fertile [3].

Diagnosis of Perrault syndrome is often based on common clinical manifestations of SNHL in females and males as well as ovarian dysfunction in females with normal karyotypes [1, 4]. Further, this diagnosis should only be made after exclusion of other potential diagnoses with symptoms similar to those of Perrault syndrome. Perrault syndrome is uncommon, and approximately 100 affected individuals have been reported to date [4]. The disease is clinically and genetically heterogeneous, and some patients show neurological signs in addition to typical deafness and premature ovarian failure [4]. Due to the complex clinical phenotype of the disease, affected males without affected sisters are diagnosed with nonsyndromic deafness rather than Perrault syndrome. The diagnosis of Perrault syndrome is confirmed by the presence of biallelic pathogenic variants in one of six genes, such as *CLPP*, *ERAL1*, *HARS2*, *HSD17B4*, *LARS2*, and *TWNK* [1, 4–9]. Currently, these genes explain approximately 40% of the causes of Perrault syndrome, but the genetic bases of more than half the cases of Perrault syndrome remain unclear [4].

Only seventeen cases of *HARS2* variants spanning nine families have been reported to date [1, 4, 10–12]. Due to its very low incidence and clinical heterogeneity, much information remains to be collected with regard to this variant. In the present study, two novel putative pathogenic variants of *HARS2* were identified in two male individuals, from the same Chinese family, with autosomal recessive non-syndromic SNHL. These two cases and a review of the literature were used to explore the correlation between phenotype and the *HARS2* genotype.

## Materials and methods

### Patients

**Fig. 1:**
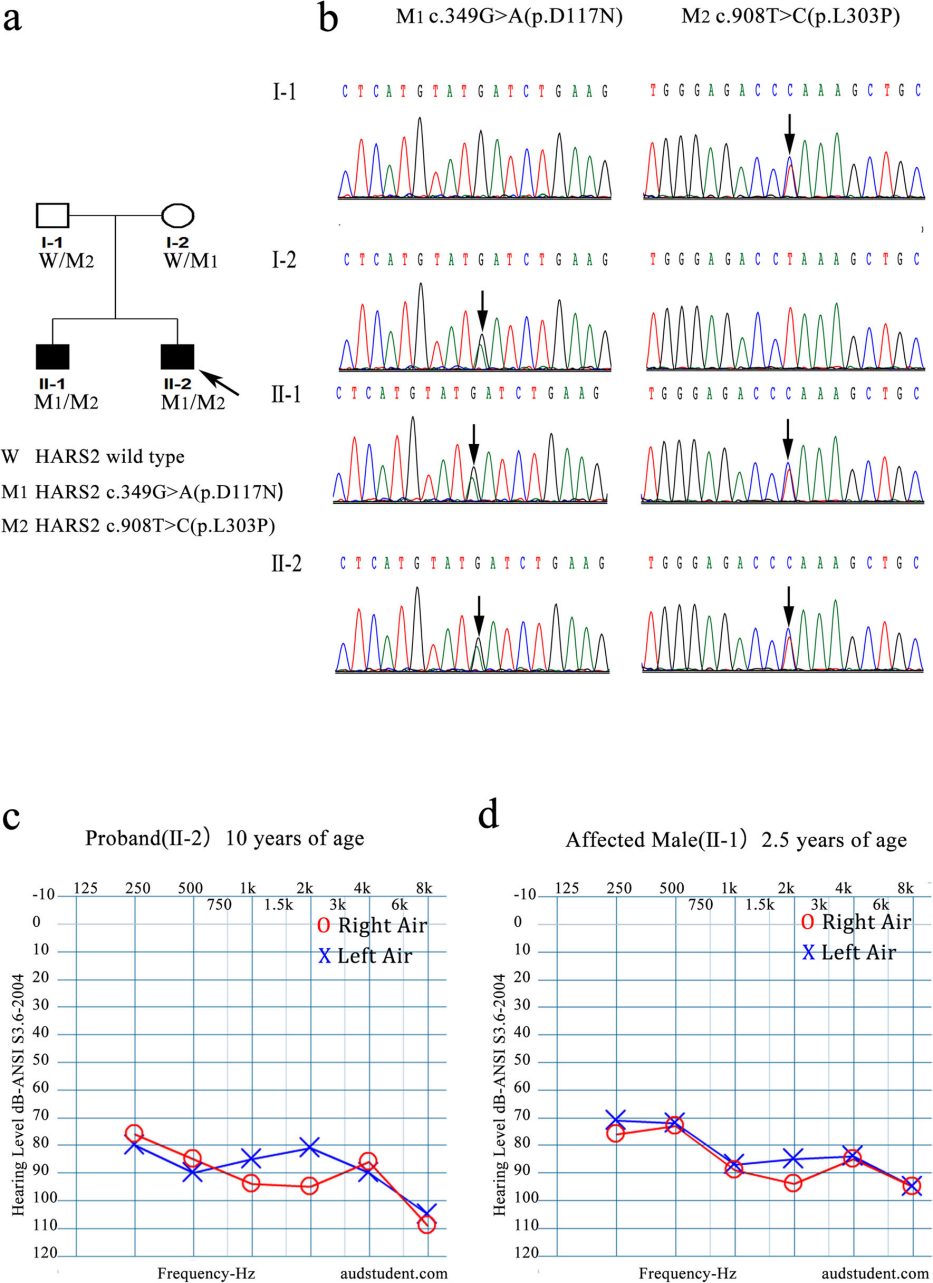
Pedigree and sequence analysis of an NSHL family. **a** The pedigree of the family. Affected individuals are denoted in black. The arrow indicates the proband. W, wild type; M_1_, *HARS2* gene NM_012208.3;c.349G > A (p.Asp117Asn); M_2_, *HARS2* gene NM_012208.3;c.908 T > C (p.Leu303Pro). **b** Sanger sequencing electropherograms of two variants. **c** Audiogram of the proband at 10 years of age. **d** Audiogram of the affected brother at 2.5 years of age

A two-generation Chinese family with SNHL was recruited for the present study. This family consisted of four members, including two siblings (II-1 and II-2, Fig. 1a) showing rapidly progressive prelingual SNHL and two normal parents (I-1 and I-2, Fig. 1a). A detailed physical examination and an audiometric assessment were performed on the two affected brothers, including ear electron microscopy, auditory brainstem response (ABR) testing, pure tone audiometry (PTA), computed tomography (CT) of the tympanic membrane and the mastoid process, and magnetic resonance imaging (MRI) of the bilateral inner ear canal and the brain. These tests were used to detect external auditory canal, middle ear, and inner ear malformations as well as auditory nerve abnormalities. The participating parents were assessed through clinical interviews.

### Targeted next-generation sequencing and data analysis

Genomic DNA was extracted from the subjects’ peripheral blood leukocytes using a Qiagen DNA Blood Kit (Qiagen, Germany) in accordance with the standard extraction protocol, and DNA integrity was detected by agar gel electrophoresis. The target regions of 139 disease-related genes in the patients’ genomic DNA were captured using an Agilent Capture Kit. An Illumina HiSeq sequencing system (Illumina, San Diego, CA, USA) was used for Next-generation sequencing of coding regions, gene regulatory regions, and 10 bp flanking introns of target genes (see Additional file 1). The deafness-related sites of the patients’ mitochondrial DNA were detected by Matrix-assisted laser Desorption Ionization-time Of Flight Mass Spectrometry (MALDI-TOF-MS), and the candidate variant sites of the family DNA samples were verified by Sanger sequencing. The sequencing data was analyzed using the Sentieon software suite, while variant annotation and screening were performed using Woxi NextCODE software (Shanghai, China).

Three major databases containing reported or potential pathogenic variants, including ClinVar, OMIM, and HGMD, were used to screen for known pathogenic variants. A population-based database of large-scale sequencing genome Aggregation Database (gnomAD), the Exome Aggregation Consortium (ExAC), 1000 genomes, and an internal whole exome sequencing (WES) database of 2114 Han Chinese were used to filter minor allele frequencies (MAFs) < 1% of the variation. Further screening for rare variants was conducted according to the interpretive guidelines of the American College of Medical Genetics and Genomics. Phenotypes of selected genes were analyzed to exclude genes not related to the patients’ clinical presentations and genetic patterns.

### Validation of variants and inheritance analysis

All candidate pathogenic variants were confirmed by Sanger sequencing. Specific primers were designed to amplify the regions containing the variants by polymerase chain reaction (PCR, see Additional file 1). The PCR products were sequenced on an ABI 3730XL Genetic Analyzer (Applied Biosystems Life Technologies) according to the manufacturer’s protocols.

### In silico analysis

A variety of tools were used to predict the pathogenicity of missense variants, including SIFT, Poly-Phen2, the Combined Annotation Dependent Depletion (CADDv1.3) score, and MutationTaster. Multiple amino acid sequence alignments of different species were conducted using ClustalW includeding *HARS2* orthologues from *Homo sapiens*, *Pan troglodytes, Macaca mulatta, Felis catus, Mus musculus, Danio rerio, Drosophila melanogaster, Caenorhabditis elegans, Bos taurus,* and *Rattus norvegicus* as well as the human *HARS* paralogue. The GERP++ score was used to evaluate evolutionary conservatism, and its value ranged from − 12.3 to 6.17, with 6.17 as the most conservative score [13]. To investigate the potential effects of each *HARS2* variant on protein conformation, the protein structure of the *HARS2* variant was predicted using the online protein model prediction server SWISS-MODEL. Analysis of protein structure changes and amino acid interactions were performed using the PyMOL Molecular Graphics System.

### Plasmid construction

Human *HARS2* cDNA sequence (NM_012208) was cloned into the pcDNA3.1-3xFlag-C vector. Using The Q5® Site-Directed Mutagenesis Kit (New England Biolabs, USA), plasmids carrying missense mutants (p.Asp117Asn or p.Leu303Pro) were constructed on the basis of wild-type plasmids and verified by sequencing.

### Western blot and immunocytochemistry assays

HEK293T cells and COS7 cells were kindly provided by the Key Laboratory for Human Disease Gene Study of Sichuan Province. The plasmid was transfected into HEK293T cells cultured in six-well plates, and the expression of HARS2 was detected by Western Blot. The plasmids were transfected into COS7 cells cultured in 24-well plates, and protein localization was detected by immunocytochemistry 48 h after transfection. Anti-FLAG (#14793, Cell Signaling Technology) is used to detect the target protein and Anti-GAPDH (#SA00001–2, Proteintech) was used as a loading control. MitoTracker Red CMXRos (#9082, Cell Signaling Technology) was used to label mitochondria. Each experiment was performed three independent determinations.

### Determination of intracellular reactive oxygen species

Intracellular reactive oxygen species (ROS) were determined by dichlorofluorescin diacetate (DCFH-DA, Beyotime, China). DCFH-DA can penetrate cell membranes and be hydrolyzed to DCFH. Intracellular ROS oxidize DCFH to fluorescent-producing DCF. Intracellular ROS levels can be measured by measuring fluorescence intensity. Briefly, the Plasmids were transfected into HEK293T cells cultured in 6-cm culture dishes, and the cells were collected 48 h later. The cells were incubated at 37 °C with 10uM DCFH-DA for 20 min, washed with PBS twice, and the fluorescence of DCF was detected by flow cytometer (BD Bioscience).

### Statistical analysis

Statistical analysis was carried out using Student unpaired double-tailed t test contained in GraphPad Prism software. *P* < 0.05 was considered to be significantly different from the control group.

## Results

### Clinical presentation

The recruited family included two affected siblings and two parents with normal phenotypes (Fig. 1a). The proband (II-2) had not passed the Universal Newborn Hearing Screening (UNHS) and presented with mild to moderate hearing loss at 1 year of age. At 3 years of age, he was diagnosed with otitis media and was treated with a bilateral tympanostomy tube. Hearing aids were provided at the age of 4 years. After the initial positive effects of hearing aids, hearing in both ears decreased with each year. When the proband was 10 years old, the audiogram showed severe bilateral SNHL (Fig. 1c), and he received a cochlear implant in his right ear. The older brother of the proband (II-1) was diagnosed with bilateral profound SNHL at the age of 2.5 years (Fig. 1d) but had previously been suspected by his parents of having a poor response to sound. He had begun to use hearing aid in both ears, which initially improved his hearing but gradually lost its effect. Therefore, he received a bilateral cochlear implant at the age of 7 years. The affected siblings underwent normal motor, cognitive, and behavioral development.

The patients’ Romberg tests were negative, and their tandem gaits were normal. An MRI of the temporal bone excluded inner ear malformations in the affected individuals. Their parents had normal hearing and were not consanguineous. A medical examination of all the family members revealed no signs of systemic disease or malformation. A survey of this family revealed no history of deafness. The inheritance pattern of the family appeared to be autosomal recessive.

### Genetic findings

The genomic DNA of the proband was analyzed using targeted next-generation sequencing. Homozygous or compound heterozygous variants were filtered for potential causing mutation. Considering de novo mutation, it is still possible that the disease shows a pattern of X-linked recessive inheritance or autosomal dominant with incomplete penetrance in this family. Hemizygous| and heterozygous variants in disease-causing genes for X-linked recessive deafness and autosomal dominant deafness were also filtered for potential causal mutation. None of variants in disease-causing genes for X-linked recessive deafness and autosomal dominant deafness was found. Two novel potentially pathogenic missense variants of *HARS2* NM_012208.3:c.349G > A (p.Asp117Asn) and c.908 T > C (p.Leu303Pro) were identified. Sanger sequencing revealed that both affected siblings carried two novel missense variants of the *HARS2* gene. Sanger sequencing confirmed that the father (I-1) was a heterozygous carrier of c.908 T > C (p.Leu303Pro) and the mother (I-2) was a heterozygous carrier of c.349G > A (p.Asp117Asn), indicating complete cosegregation of the variants with the disease phenotype in this family (Fig. 1b).

### In silico analysis of the variants detected in HARS2

**Fig. 2:**
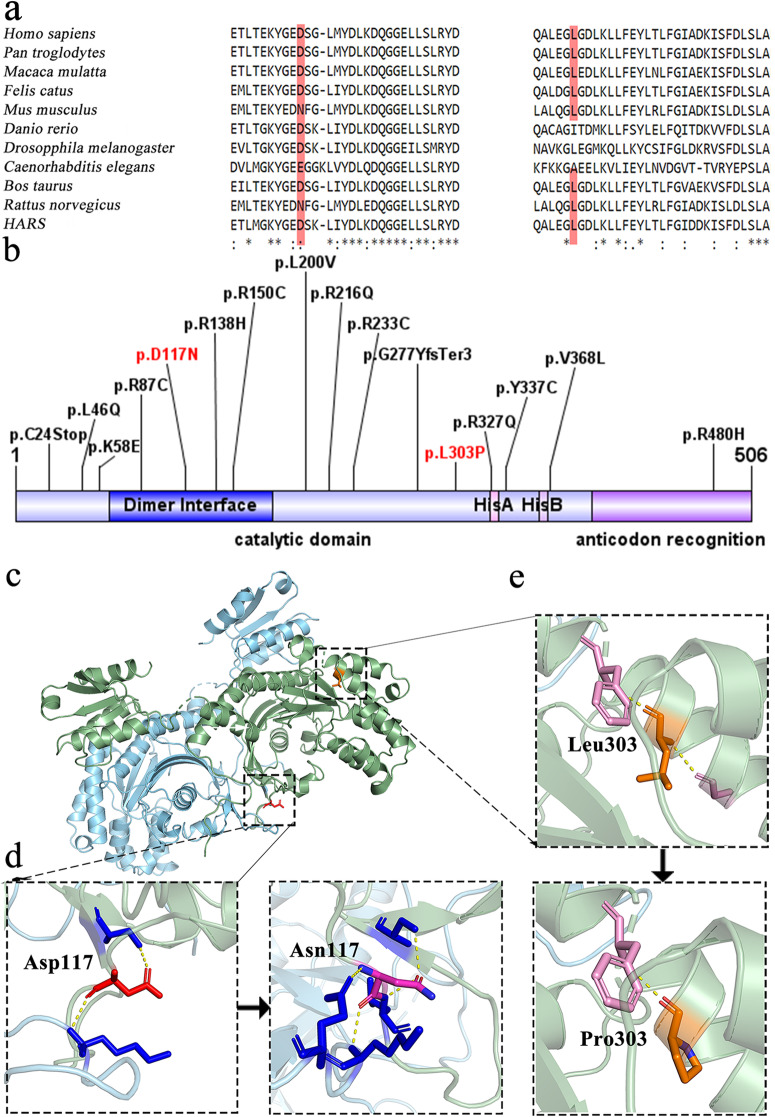
In silico analysis of *HARS2* variants*.* **a** Multispecies sequence alignment of the *HARS2* protein showing the evolutionary conservation of two mutated amino acids (p.Asp117Asn and p.Leu303Pro). Red shading indicates amino acids shared among all eight species. **b** Newly identified and previously reported *HARS2* variants are marked along the schematic representation of *HARS2*. The N-terminal catalytic domain (amino acid; aa 1–405) and the C-terminal anticodon binding domain (aa 406–506). The catalytic domain contains the highly conserved dimer interface shown in dark blue (aa 65–177) and the histidine recognition and binding sites shown in pink (HisA: aa 327–332 and HisB: aa 361–365) **c** Crystal-structure-based in silico modeling of a dimeric *HARS2* protein. This 3D model of the *HARS2* protein is based on the structure of dimeric human HisRS (PDB ID 4phc). Two monomers of the homodimer are shown in green and blue, respectively. The Asp117 residue is located in the s1 loop at the dimer interface, as indicated by the red stick. The Leu303residue is located in an α-helix displayed in orange. **d** Close-up view of residue 117 and other residues that interacted. **e** Close-up view of residue 303 and other residues that interacted

The c.349G > A substitution results in the substitution of asparticacid by asparagine (p.Asp117Asn). The c.908 T > C results in a single amino acid substitution: leucine to proline (p.Leu303Pro). Neither of these variants was found in the ExAC or in-house WES databases, but they were found in the heterozygous state from gnomAD (Table 1). The multiple amino acid sequence alignment of *HARS2* showed that the two mutant residue sites were evolutionally conserved in nine species (Fig. 2a). Both variants were located in the catalytic domain of *HARS2*, in accordance with previously reported potential pathogenic variants of *HARS2* (Fig. 2b). These two variants were predicted to be deleterious by most of the in silico software programs (CADD, Poly-Phen2, MutationTaster, and SIFT), further supporting variant pathogenicity (Table 1).

**Table 1:** Bioinformatics analysis of presently identified *HARS2* variants and variants that have been previously associated with Perrault syndrome or hearing loss

ID	Mutation	gnomAD	CADD	SIFT	Polyphen-2	Mutationtster	GERP++	Pathogenicity Classification	References
1	c.72C > A(p. Cys24Stop)	0	24	Deleterious (0)	Damaging (1)	Disease Causing (1)	Yes (5.27)	Likely Pathogenic	[11]
2	c.137 T > A (p.Leu46Gln)	0	23.8	Tolerated (0.1)	Damaging (1.0)	Disease Causing (1)	Yes (4.07)	Uncertain Significance	[12]
3	c.172A > G(p.Lys58Glu)	0.000006	26.9	Deleterious (0)	Damaging (0.99)	Disease Causing (1)	Yes (5.96)	Uncertain Significance	[12]
4	c.259C > T(p.Arg87Cys)	0.00001	31	Deleterious (0)	Damaging (0.94)	Disease Causing (1)	Yes (5.3)	Uncertain Significance	[12]
5	c.349G > A (p.Asp117Asn)	0.00000397	23.9	Tolerated(0.12)	Benign(0.186)	Disease Causing (1)	Yes (5.65)	Likely Pathogenic	This study
6	c.413G > A (p.Arg138His)	0.000028	34	Deleterious (0)	Damaging (1)	Disease Causing (1)	Yes (6.17)	Uncertain Significance	[11]
7	c.448C > T(p.Arg150Cys)	0.00005	32	Deleterious (0)	Damaging (1)	Disease Causing (1)	Yes (5.3)	Likely Pathogenic	[12]
8	c.598C > G(p.Leu200Val)	0.00003	26.7	Deleterious (0)	Damaging (1)	Disease Causing (1)	Yes (4.38)	Likely Pathogenic	[1]
9	c.647G > A(p.Arg216Gln)	0.00000797	23.4	Tolerated(0.15)	Damaging(0.989)	Disease Causing (1)	Yes (5.18)	Uncertain Significance	[10]
10	c.697C > T(p.Arg233Cys)	0	33	Deleterious(0)	Damaging(0.999)	Disease Causing (1)	Yes (5.3)	Uncertain Significance	[10]
11	c.828delTinsGTATCCCTAGTATTTCTACTA (p.Gly277TyrfsTer3)	0	/	/	/	/	/	Likely Pathogenic	[11]
12	c.908 T > C (p.Leu303Pro)	0.000008	24.2	Deleterious (0)	Benign(0.047)	Disease Causing (1)	Yes (5.78)	Likely Pathogenic	This study
13	c.980G > A(p.Arg327Gln)	0.000004	34	Deleterious (0)	Damaging (1)	Disease Causing (1)	Yes (3.95)	Uncertain Significance	[12]
14	c.1010A > G(p.Tyr337Cys)	0.000004	28.3	Deleterious (0)	Damaging (1)	Disease Causing (1)	Yes (5.67)	Uncertain Significance	[4]
15	c.1102G > T(p.Val368Leu)	0.000008	25.9	Deleterious (0)	Damaging (0.99)	Disease Causing (1)	Yes (5.67)	Likely Pathogenic	[1]
16	c.1439G > A(p.Arg480His)	0.000074	35	Deleterious (0)	Damaging (1)	Disease Causing (1)	Yes (5.5)	Likely pathogenic	[11]

*HARS2*; NM_012208.3. Output prediction of each tool-CADD defined scores ranging from 1 to 99 based on the rank of each variant relative to all possible 8.6 billion substitutions in the human reference genome. Reference genome single nucleotide variants at the top 10% of CADD scores are assigned to CADD-10, those as the top 1% are assigned to CADD-20, those at the top 0.1% are assigned to CADD-30, and so on. SIFT: tolerated, damaging. Polyplen-2: benign, possibly damaging, probably damaging. Taster mutation: disease-causing, polymorphism. GERP++ scores ranged from −12.3 to 6.17, with 6.17 being the most conservative score. Pathogenicity classification: According to the ACMG scoring rules, the scoring classifications were pathogenic, likely pathogenic, uncertain significance, likely benign, and benign

A homologous *HARS2* model was built by SWISS-MODEL (Fig. 2c). The results showed that Asp117 residue was located in the s1 loop at the dimer interface and formed a hydrogen bond with the Lys118 residue on another monomer. The substitution of the Asp residue with an Asn residue increased interaction with the Gln121 and Ser127 residues (Fig. 2d). The Leu303 residue was predicted to be located in a α-helix, and the substitution of Pro for Leu lost the hydrogen bond between Leu303 and Gly297. this may have affected the stability of crystal conformation (Fig. 2e).

### HARS2 mutation results in elevated intracellular ROS production

**Fig. 3:**
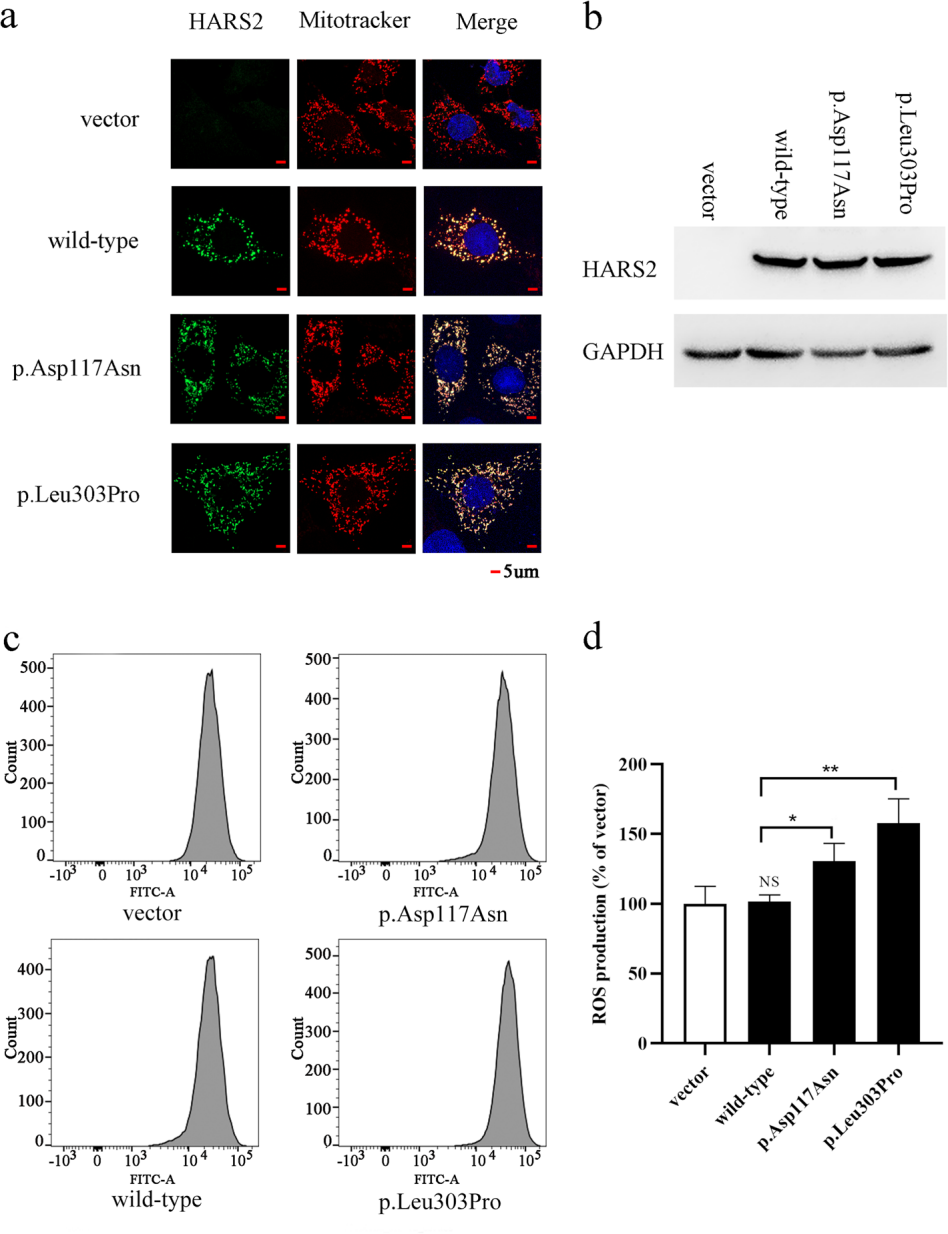
The mutant plasmid increased ROS production in HEK293T cells, but did not affect protein expression and localization. **a** The protein localization of mutant *HARS2* is similar to that of wild *HARS2*, both of which are located in mitochondria. **b** With *GAPDH* as internal control, there was no significant difference in the expression levels of wild-type and mutant proteins. **c** Representative flow cytometry peaks of HEK293T cells were transiently transfected with empty vector, wild-type *HARS2* and mutant *HARS2* (p.Asp117Asn or (p.Leu303Pro) plasmid for 48 h, followed by flow cytometry analysis to detect intracellular ROS production. **d** The relative ratio of intensity was calculated. The average of three independent determinations for each treatment is shown

To determine the effects of *HARS2* variants (p.Asp117Asn and p.Leu303Pro) on protein expression and localization, Western blot and Immunohistochemical analysis were performed. The results show that these two missense mutations do not change the expression and subcellular localization of HARS2 protein (Fig. 3a and b). To explore the effect of *HARS2* gene variants on mitochondrial function, we performed intracellular ROS detection on HEK293T cells transfected with wild-type and mutant plasmids. Our results showed that *HARS2* p.Asp117Asn and p.Leu303Pro mutations increased the production of ROS in HEK293T cells compared with cells transfected either with wild-type *HARS2* or control empty vectors (Fig. 3c and d). The results suggested that these two missense mutations may alter its function and promote the increased production of ROS.

## Discussion

In the present study, we identified two novel missense variants—c.349G > A (p.Asp117Asn) and c.908 T > C (p.Leu303Pro)—in the *HARS2* genes of two male individuals with NSHL from the same Chinese family. Both p.Asp117Asn and p.Leu303Pro are highly conserved across species and located in the catalytic domain, which is the critical domain of mitochondrial histidyl tRNA synthetase. These two variants were found at extremely low frequencies in the control databases. Functional assay have shown that the two mutations do not affect protein expression and localization. However, the two variants results in elevated intracellular ROS production. In most mammalian cells, ROS is mainly produced by mitochondria, and mitochondrial dysfunction leads to the increased ROS production [14, 15]. Excessive accumulation of ROS in cochlear cells and subsequent apoptosis induction have been implicated in the development of sensorineural hearing loss [15]. Therefore, in accordance with the guidelines of the American College of Medical Genetics and Genomics for sequence variant interpretation [16], c.349G > A (p.Asp117Asn) and c.908 T > C (p.Leu303Pro) were interpreted as likely pathogenic variants.

To date, 16 variants of *HARS2* have been associated with deafness or Perrault syndrome, including c.908 T > C (p.Leu303Pro) and c.349G > A (p.Asp117Asn) in the present study (Table 1). The *HARS2* protein is a homodimer enzyme belonging to the class II family of aminoacyl-tRNA synthetases. *HARS2* protein prediction consists of two domains: (1) an N-terminal catalytic domain, including a dimer interface and a HisRS-specific helical domain that binds to the acceptor stem of tRNA; and (2) a C-terminal domain, which is involved in recognition of the anticodon stem and loop of tRNA. Fifteen variants are located in the catalytic domain of *HARS2*, only p.Arg480His lies in the C-terminal anticodon binding domain (Fig. 2b).

Mutations in *HARS2* genes resulted in diseases with significant clinical heterogeneity not only in these families but also in individual family members (Table 2). The typical clinical phenotype of Perrault syndrome was observed in four families previously reported with biallelic *HARS2* variants [1, 4, 11, 12]. Clinical features of Perrault syndrome include early or late onset of mild to profound bilateral sensorineural deafness as well as irregular menstruation, secondary amenorrhea, premature ovarian failure, and other manifestations of ovarian dysfunction in women. It is worth noting that it has been reported that only non-syndromic deafness phenotype was found in female members of Perrault syndrome family, while her sister showed irregular menstruation [12]. Perrault syndrome may be latent in some families with non-syndromic deafness associated with *HARS2* mutations. The definitive diagnosis of Perrault syndrome based on clinical features alone is a challenge in sporadic males, and preadolescent females with no signs of POI.

**Table 2:** Clinical features of all reported patients with *HARS2* variants

Patient		Origin/ Descent	HARS2 Variants		Sex	Age of Onset	Age at Exam	Degree of hearing loss	Hearing habilitation	Gonadal dysfunction	Age at POI	Neurological features	Additional features	Reference
			M1	M2										
Family 1	II-1	European	c.598C > G p.Leu200Val	c.1102G > T p.Val368Leu	F	34	NR	Mild	NR	ovarian dysgenesis, with amenorrhea and streak gonads	NR	NO	NO	Pierce et.al [1]
	II-2				M	6	22	Moderate to severe	NR	NA	NA	NO	NO	
	II-4				M	18	NR	Moderate to severe	NR	NA	NA	NO	NO	
	II-7				F	12	19	Mild to moderate	NR	ovarian dysgenesis, with amenorrhea and streak gonads	NR	NO	NO	
	II-8				F	8	13	Moderate to profound	NR	ovarian dysgenesis, with amenorrhea and streak gonads	NR	NO	NO	
Family 2	IV-1	Morocco	c.1010A > G p.Tyr337Cys	(Hom)	F	< 3	NR	Profound	NR	Secondary Amenorrhea	25	NO	Hypothyroidism	Lerat et.al [4]
	VI-1				F	< 3	NR	Profound	NR	Secondary Amenorrhea	26	NO	Hypothyroidism	
Family 3	proband	Chinese Han	c.647G > A p.Arg216Gln	c.697C > T p.Arg233Cys	M	NR	NR	NR	NA	NA	NO	NO	NO	Zou et.al [10]
Family 4	Proband	European	c.137 T > A p.Leu46Gln	c.259C > T p.Arg87Cys	F	2.5	32	Moderate	Bilateral CI	Secondary amenorrhea, cessation	24	NO	NO	Karstensen et.al [12]
Family 5	II-1	European	c.172A > G p.Lys58Glu	c.448C > T p.Arg150Cys	M	5	18	Moderate to severe	Bilateral CI	NA	NA	NO	NO	Karstensen et.al [12]
	II-2				F	2.6	16	Moderate to severe	Bilateral CI	irregular menses	16	NO	NO	
	II-3				F	0.3	13	Moderate to severe	Bilateral CI	asymptomatic	13	NO	NO	
Family 6	Proband	European	c.448C > T p.Arg150Cys	c.980G > A p.Arg327Gln	F	2	7	Moderate to severe	Bilateral CI	asymptomatic	7	NO	NO	Karstensen et.al [12]
Family 7	Proband	European	c.413G > A p.Arg138His	c.1439G > A p.Arg480His	F	6	6	Moderate	HA	asymptomatic	NR	NO	NO	Demain et.al [11]
Family 8	II-1	North American	c.828delTinsGTATCCCTAGTATTTCTACTA p.Gly277TyrfsTer3	c.1439G > A p.Arg480His	M	2.5	14	Severe to profound	CI + HA	NA	NA	Difficulty with fine motor movements	Problems with tooth growth	Demain et.al [11]
	II-2				M	1	11	Severe to profound	CI + HA	NA	NA	Tightness in the muscles of his lower limbs	Problems with tooth growth	
Family 9	Proband	North American	c.72C > A p.Cys24Stop	c.1439G > A p.Arg480His	M	1.5	4	Profound	Not recorded	NA	NA	NO	NO	Demain et.al [11]
Family 10	II-1	Chinese Han	c.349G > A p.Asp117Asn	c.908 T > C p.Leu303Pro	M	2.5	7	Profound	HA + Right CI	NA	NA	NO	NO	This study
	II-2				M	1	10	Severe to profound	HA + CI	NA	NA	NO	NO	

*HA* Hearing aid, *CI* Cochlear implant, *POI* Primary ovarian insufficiency, *F* Female, *M* Male, *NA* Not applicable, *NR* Not recorded

Previous studies have shown that the missense mutations in *HARS2* do not affect protein expression but significantly impact enzyme activity [1], resulting in decreased levels of aminoacylated tRNA His. Gong et al. [17] found that the deafness-associated mitochondrial tRNA^His^ 12,201 T > C mutation also decrease in the steady-state level of tRNA^His^ and further alters the mitochondrial function by affecting mitochondrial protein translation, Due to reduction ATP synthesis and respiratory defects, decreased mitochondrial membrane potential, impaired oxidative phosphorylation, and increased ROS [17]. Overexpression of *HARS2* in cells could partially rescue the above mitochondrial defects [18]. In the present study, two variants c.908 T > C (p.Leu303Pro) and c.349G > A (p.Asp117Asn) increased intracellular ROS levels, suggesting that mitochondrial function might be damaged. we speculate that missense mutations of *HARS2* may impair protein function, and its pathogenic mechanism may be related to mitochondrial functional defects. *HARS2* is highly expressed in the cochlea and the neural tube [19]. The cochlea is particularly sensitive to mitochondrial dysfunction. Further functional experiments are needed to elucidate the pathogenesis of *HARS2* variants.

In conclusion, we described a rare pedigree of Perrault syndrome. Two novel putative pathogenic variants of *HARS2* were identified in two affected male individuals. These novel variants further expanded the existing spectrum of *HARS2* variants and phenotypes of Perrault syndrome or SNHL, which can assist in molecular diagnosis and genetic counselling of patients with SNHL. The correlation between phenotype and *HARS2* genotype was explored alongside preexisting clinical reports. The present cohort was too small to elucidate a relation between genotypes and phenotypes, as 16 variants of *HARS2* have been associated with deafness or Perrault syndrome in 10 cases to date. A series of studies will be performed in the future to explore phenotype-genotype correlations within a larger sample.

## Supplementary Information

**Additional file 1: Table S1.** Gene list in the panel performed by target sequencing of the exons and mutations associated with deafness in mitochondrial DNA. **Table S2.** The sequences of polymerase chain reaction (PCR) primers and PCR product sizes.

## Data Availability

The data sets generated during and analyzed during the current study are available from the corresponding author on reasonable request.

## Abbreviations

SNHL: Sensorineural hearing loss
POI: Primary ovarian insufficiency
