# Supplementary material for: Two novel likely pathogenic variants of HARS2 identified in a Chinese family with sensorineural hearing loss
Source: Hereditas. 2020 Nov 24;157:47. doi: 10.1186/s41065-020-00157-7 (PMC7684720; doi:10.1186/s41065-020-00157-7)
Supplement: Supplementary file 1 — Additional file 1: Table S1. Gene list in the panel performed by target sequencing of the exons and mutations associated with deafness in mitochondrial DNA. Table S2. The sequences of polymerase chain reaction (PCR) primers and PCR product sizes. [file 41065_2020_157_MOESM1_ESM.doc]

**Supplementary Information**

Additional file 1:Table s1 Gene list in the panel performed by target sequencing of the exons and mutations associated with deafness in mitochondrial DNA.

Additional file 1:Table s2 The sequences of polymerase chain reaction (PCR) primers and PCR product sizes.

Table s1 Gene list in the panel performed by target sequencing of the exons and mutations associated with deafness in mitochondrial DNA.

| *ABHD12* | *COCH* | *EYA1* | *KARS* | *NDP* | *RMND1* | *SUCLA2* |
| --- | --- | --- | --- | --- | --- | --- |
| *ACTG1* | *COL11A1* | *EYA4* | *KCNE1* | *NF2* | *RPS6KA3* | *SUCLG1* |
| *AIFM1* | *COL2A1* | *FGF3* | *KCNJ10* | *NLRP3* | *SALL4* | *TBC1D24* |
| *ALMS1* | *COL4A3* | *FOXI1* | *KCNQ1* | *OPA1* | *SEMA3E* | *TCOF1* |
| *ANKH* | *COL4A4* | *GAA* | *KCNQ4* | *OTOA* | *SERPINB6* | *TECTA* |
| *ATP6V1B1* | *COL4A5* | *GALNS* | *LHFPL5* | *OTOF* | *SIX1* | *TFAP2A* |
| *BCS1L* | *COL4A6* | *GATA3* | *LOXHD1* | *OTOG* | *SIX5* | *TIMM8A* |
| *BSND* | *COL9A1* | *GIPC3* | *LRP2* | *OTOGL* | *SLC17A8* | *TJP2* |
| *BTD* | *COL9A2* | *GJB2* | *LRTOMT* | *PAX3* | *SLC19A2* | *TMC1* |
| *CABP2* | *COL9A3* | *GJB3* | *MANBA* | *PCDH15* | *SLC26A4* | *TMIE* |
| *CACNA1D* | *GSDME* | *GJB6* | *MARVELD2* | *PDZD7* | *SLC26A5* | *TMPRSS3* |
| *CCDC50* | *WHRN* | *ADGRV1* | *MET* | *PHYH* | *SLC29A3* | *TPRN* |
| *CDH23* | *PJVK* | *GPSM2* | *MITF* | *PNPT1* | *SLC33A1* | *TRIOBP* |
| *CEACAM16* | *DIABLO* | *GRHL2* | *MSRB3* | *POLR1C* | *SLC52A2* | *TSPEAR* |
| *CHD7* | *DIAPH1* | *GRXCR1* | *MYH14* | *POLR1D* | *SLC52A3* | *TYR* |
| *CHSY1* | *DSPP* | *HARS* | *MYH9* | *POU3F4* | *SMAD4* | *USH1C* |
| *CIB2* | *EDN3* | *HARS2* | *MYO15A* | *POU4F3* | *SMPX* | *USH1G* |
| *CISD2* | *EDNRB* | *HOXB1* | *MYO3A* | *PRPS1* | *SNAI2* | *USH2A* |
| *CLDN14* | *ESPN* | *HSD17B4* | *MYO6* | *PTPRQ* | *SOX10* | *WFS1* |
| *CLRN1* | *ESRRB* | *ILDR1* | *MYO7A* | *RDX* | *STRC* |  |
| m.C1494T | m.A1555G | m.A7445G | m.A7445C | m.T7510C | m.T7511C |  |

Table s2 The sequences of polymerase chain reaction (PCR) primers and PCR product sizes.

| Primer name | Forward | Reverse | Size |
| --- | --- | --- | --- |
| Yc-hars2-c349 | TTCTCATCTGTGTTTTGGAGTCAT | CCCGCTGGATTTGGAAAG | 208 |
| Yc-hars2-c908 | TGTGAGTGAACAGCAGAGACTTTAT | AGCCTGAGTTGGGGTCTGC | 346 |
